# Role of the tumor microenvironment in pancreatic cancer

**DOI:** 10.1002/ags3.12225

**Published:** 2019-01-04

**Authors:** Takashi Murakami, Yukihiko Hiroshima, Ryusei Matsuyama, Yuki Homma, Robert M. Hoffman, Itaru Endo

**Affiliations:** ^1^ Department of Gastroenterological Surgery Yokohama City University Graduate School of Medicine Yokohama Japan; ^2^ AntiCancer, Inc. San Diego California; ^3^ Department of Surgery University of California San Diego California

**Keywords:** immune cell, immunomodulation, pancreatic cancer, tumor‐infiltrating lymphocyte, tumor microenvironment

## Abstract

Pancreatic cancer remains a highly recalcitrant disease despite the development of systemic chemotherapies. New treatment options are thus urgently required. Dense stromal formation, so‐called “desmoplastic stroma,” plays controversial roles in terms of pancreatic cancer growth, invasion, and metastasis. Cells such as cancer‐associated fibroblasts, endothelial cells, and immune cells comprise the tumor microenvironment of pancreatic cancer. Pancreatic cancer is considered an immune‐quiescent disease, but activation of immunological response in pancreatic cancer may contribute to favorable outcomes. Herein, we review the role of the tumor microenvironment in pancreatic cancer, with a focus on immunological aspects.

## 1 INTRODUCTION

Patients with pancreatic cancer show a dismal prognosis, with 5‐year overall survival rates of 7%‐8% in both Japan and the USA.^1^ Pancreatic‐cancer death is estimated to become the second most common cause of cancer death by 2030 in the USA.^2^ A lack of symptoms or biomarkers in the early stages of the cancer, an aggressive biological feature in that cancer cells metastasize to distant lesions even from small tumors, and drug resistance as a result of dense fibrous stroma all contribute to poor treatment outcomes of pancreatic cancer. Furthermore, pancreatic cancer is known to create an immune‐suppressive microenvironment that results in immune evasion from the host antitumor immune system, leading to rapid cancer progression. Recent studies have shown that the tumor microenvironment of pancreatic cancer, including cancer‐associated fibroblasts such as stellate cells, extracellular matrix, various kinds of immune cells, and cytokines released by these cells, participates in controlling tumor growth, invasion, and metastasis by means of close interactions with cancer cells. Hence, preclinical and clinical studies have focused on the tumor microenvironment as a potential novel target that may lead to cure for pancreatic cancer.

**Figure 1:**
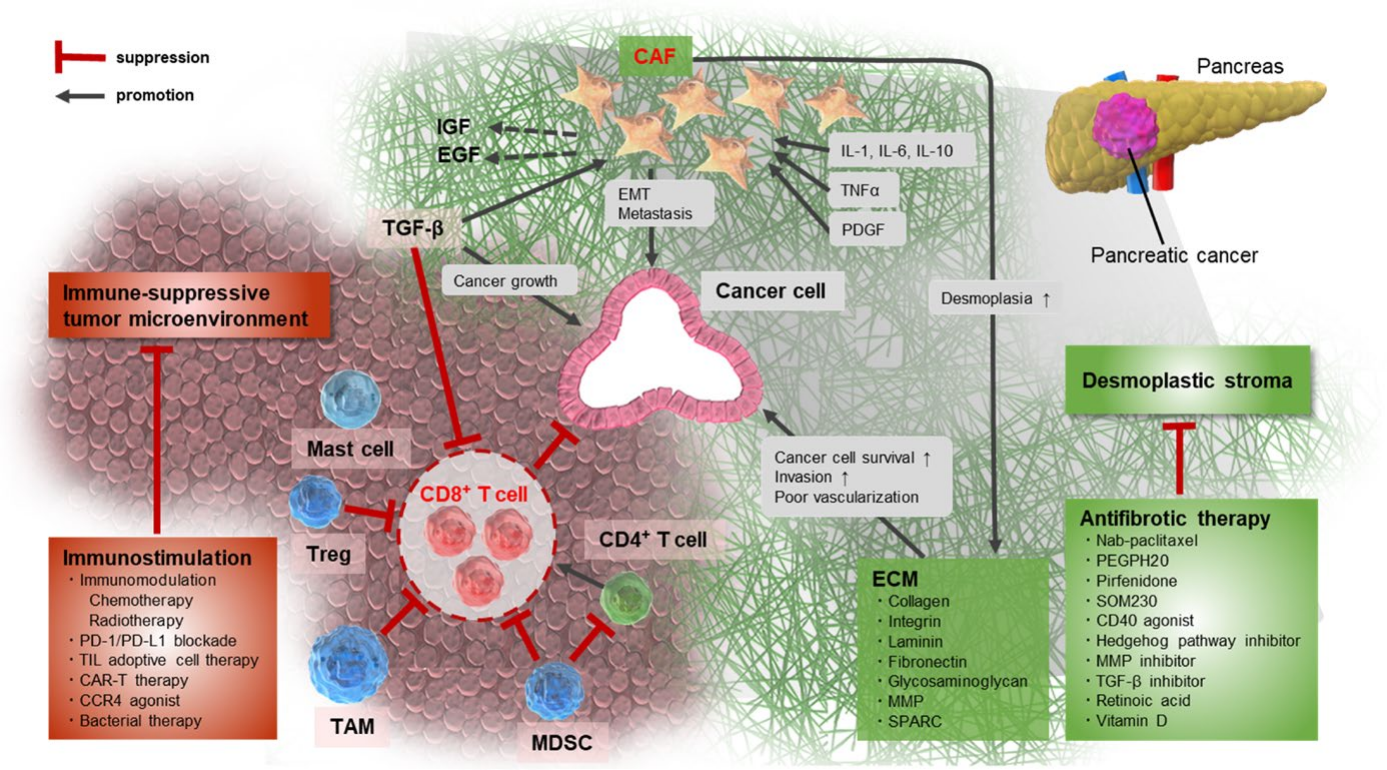
Schematic of the tumor microenvironment in pancreatic cancer. The tumor microenvironment in pancreatic cancer contributes to tumor progression in a multifaceted way. Cancer‐associated fibroblasts (CAFs) and the extracellular matrix (ECM) comprise the desmoplastic stroma and enhance cancer growth, invasion, and metastasis in direct or indirect ways. In contrast, immune‐suppressor cells such as regulatory T cells (Treg), myeloid‐derived suppressor cells (MDSC), and tumor‐associated macrophages (TAM) inhibit CD8^+^ T cells, which play a key role in the antitumor immune response, by establishing an immunosuppressive tumor microenvironment. Cytokines secreted by CAFs, immune cells, or other components mediate these processes. Antifibrotic therapy, immunotherapy, induction of immunomodulation, and bacterial therapy may improve the unfavorable tumor microenvironment associated with pancreatic cancer. CAR‐T, chimeric antigen receptor T cell; CCR4, chemokine receptor type 4; EGF, epidermal growth factor; EMT, epithelial mesenchymal transition; IGF, insulin‐like growth factor; IL, interleukin; MMP, matrix metalloproteinase; PD‐1, programmed cell death protein 1; PDGF, platelet‐derived growth factor; PD‐L1, programmed cell death ligand 1; PEGPH20, pegvorhyaluronidase alfa; SPARC, secreted protein acidic and rich in cysteine; TGF‐β, transforming growth factor β; TIL, tumor‐infiltrating lymphocyte; TNF‐α, tumor necrosis factor α

The present report reviews the pancreatic tumor microenvironment from the perspective of each stromal component, particularly immune cells. A potential breakthrough therapy targeting the microenvironment is discussed. Figure 1 shows a graphical abstract.

## 2 ROLE OF DESMOPLASTIC STROMA IN THE DEVELOPMENT OF PANCREATIC CANCER

Desmoplastic stroma consists of stromal cells and extracellular matrix. The fibroblastic population may comprise 90% of the whole tumor mass of pancreatic cancer.^3^ Wu et al^4^ reported that the extent of stroma can offer a prognostic factor for patients with solid cancers.

The extracellular matrix consists of a variety of materials such as collagen, integrin, laminin, fibronectin, glycosaminoglycan, matrix metalloproteinase (MMP), and secreted protein acidic and rich in cysteine (SPARC).^5^ Under normal circumstances, the extracellular matrix conserves cellular polarity, proliferation, and migration while inhibiting dysplasia.^6^ In contrast, dysregulated integrin subunits, seen in the basement membrane in pancreatic cancer tissue, contribute to cancer‐cell survival and invasiveness.^7^, ^8^ Hyaluronan, a glycosaminoglycan, is deposited in high concentration in the extracellular matrix of pancreatic cancer.^9^ Once hyaluronan binds to its receptor, CD44, subsequent interactions prolong cancer‐cell survival and promote cancer cell growth.

Stromal cells in pancreatic cancer comprise cancer‐associated fibroblasts (CAFs), endothelial cells, and immune cells. Pancreatic stellate cells are a subset of CAFs.^10^ CAFs are a major component of pancreatic cancer stroma, derived from different kinds of progenitor cells such as fibroblasts, pancreatic stellate cells, and epithelial, endothelial, and mesenchymal stem cells.^11^, ^12^ CAFs express α‐smooth muscle actin (α‐SMA), a well‐known and reliable marker of CAF, stromal cell‐derived factor‐1α, fibroblast activation protein, and fibroblast specific protein‐1.^3^, ^11^ CAFs are activated by transforming growth factor β (TGF‐β), sonic hedgehog, tumor necrosis factor α (TNF‐α), platelet‐derived growth factor (PDGF), and interleukin (IL)‐1, ‐6, and ‐10.^11^, ^13^ TGF‐β regulates tumor growth, differentiation, and immune cell function.^14^ TGF‐β initially plays a tumor‐suppressive role, but enhances tumor growth as cancer progresses.^6^, ^15^ TGF‐β1 enhances the ability of CAFs to form abundant filopodia, which allows CAFs to migrate into cancer cell nests.^13^ CAFs are stimulated by several types of mediators such as C‐C motif chemokine ligand 2 (CCL2), hepatocyte growth factor (HGF), and fibroblast growth factor (FGF).^11^ However, some growth factors including insulin‐like growth factor (IGF), epidermal growth factor (EGF), and TGF‐β are derived from CAFs.^6^, ^14^ CAFs induce desmoplasia through the secretion of collagen types I and III, fibronectin, proteoglycans, and glycosaminoglycans, leading to increased mechanical pressure in the extracellular matrix, which may promote cancer‐cell migration and inhibit vascularization.^3^ CAFs provide cancer cells with nourishment under low‐glucose conditions.^11^ CAFs also contribute to epithelial‐to‐mesenchymal transition (EMT), cancer invasion, angiogenesis, and metastasis.^16^, ^17^

## 3 ROLE OF IMMUNE CELLS IN THE TUMOR MICROENVIRONMENT OF PANCREATIC CANCER

### 3.1 Immune‐suppressive tumor microenvironment in pancreatic cancer

Pancreatic cancer is thought to be immune‐quiescent, as a variety of immune‐suppressive mechanisms can inhibit antitumor immunity.^18^ Low expression of major histocompatibility complex (MHC) class I molecules on pancreatic cancer cells inhibits T‐cell activation.^19^ CD8^+^ T cells are activated by interaction with antigen presented by MHC class I molecules on antigen‐presenting cells. Cytotoxic T cells, representing activated CD8^+^ T cells, attack cancer cells by secreting perforin and granzyme and expressing Fas ligand. CD8^+^ T cells also express immune checkpoint molecules, which restrain T‐cell functions, inducing immune exhaustion. CD8^+^ T cells play a central role in eliciting antitumor immune responses, but their function in the tumor microenvironment is impaired as a result of several immune‐suppressing mechanisms. Neoantigens show high immunogenicity arising from genetic mutations present in cancer cells.^20^ These oligopeptides of eight to 12 amino acids are presented on MHC class I receptors. Neoantigens are expressed by most pancreatic cancers, but effective immune responses fail to be generated, probably due to the immunosuppressive tumor microenvironment.^21^

Transforming growth factor β excreted by pancreatic cancer cells or extracellular matrix also restrains immune cell function.^22^, ^23^ Cancer cell‐derived indoleamine 2,3‐dioxygenase, a tryptophan‐metabolizing enzyme, results in effector T cells becoming deficient in tryptophan, inducing immunological tolerance.^24^ In a mouse model, major immune‐suppressor cell lines including regulatory T cells (Tregs), myeloid‐derived suppressor cells (MDSCs), and tumor‐associated macrophages (TAMs) have been seen in pancreatic tissue even in the early stages of carcinogenesis.^25^ Tregs were identified as CD4^+^ CD25^+^ immune‐suppressive cells in 1995.^26^ The transcriptional factor Foxp3 was shown to be a master regulator of Treg function in 2003.^27^ Tregs comprise 5%‐10% of peripheral CD4^+^ T cells in healthy hosts, whereas higher concentrations of Tregs were reported in patients with cancers, including pancreatic cancer.^28^, ^29^ Tregs maintain immune cell homeostasis by controlling self‐reactive T cells. The immune‐suppressing mechanisms induced by Tregs are as follows: suppression of effector T cells by secreting immune‐suppressive cytokines such as TGF‐β or competing for IL‐2; induction of effector T‐cell apoptosis by cytotoxic enzymes such as granzyme B; and inhibition of dendritic cell maturation and function.^30^ In pancreatic cancer tissue, abundant Tregs are present.^6^ Cytotoxic T‐lymphocyte‐associated protein 4 (CTLA‐4), which is constantly expressed on Tregs, plays a central role in suppressing antigen‐presenting cells. MDSCs induce immune evasion by inhibiting both innate and adaptive antitumor immunity in pancreatic cancer.^21^ Pancreatic‐cancer patients with higher levels of circulating MDSCs correlated with poorer overall survival.^31^ TAMs are macrophages that comprise a major component of immune cells in the tumor microenvironment. TAMs contribute to immune suppression as well as promoting angiogenesis. Cytokines such as CC chemokine, a protein belonging to the CXC chemokine family called stromal‐derived factor 1, and vascular endothelial growth factor attract TAMs into the tumor microenvironment.^32^ TAMs support cancer progression by secreting a variety of growth factors.^33^ IL‐10 secreted from TAMs contributes to establishment of the immunosuppressive tumor microenvironment by preventing dendritic cell‐mediated antitumor immune responses.^34^ Peranzoni et al^35^ reported that macrophages inhibit CD8^+^ tumor‐infiltrating lymphocytes (TIL).

### 3.2 Tumor‐infiltrating immune cells in pancreatic cancer

Cancer cells are surveyed by the host immune system, which eliminates cancer in the first phase. Cancer and immunity are then balanced in the next phase. In the last immune‐evasion phase, cancer appears in the human body. This theory of immunoediting was developed only recently.^36^ TILs are considered to reflect immunoediting.^37^ TILs are observed in several tumor types, including colorectal cancer, gastric cancer, hepatocellular carcinoma, bile duct cancer, and pancreatic cancer, which are reported to have prognostic value.^38^ Regarding pancreatic cancer, Fukunaga et al^39^ first reported that both CD4^+^ and CD8^+^ TILs are associated with longer postoperative survival. Hwang et al^40^ showed that the Foxp3^+^/granzyme B^+^ ratio correlated with both disease‐free and overall survival for patients with left‐sided pancreatic cancer. Furthermore, recent studies have shown that mast cells also affect tumor growth by enhancing angiogenesis, cancer‐cell proliferation and invasion.^41^ Mast‐cell infiltration of tumors predicts poor long‐term outcomes for colorectal cancer, hepatocellular carcinoma, colorectal cancer liver metastasis, and pancreatic cancer patients.^42^, ^43^, ^44^ Kato et al^45^ showed that semaphorin‐4D (Sema4D), a membrane‐bound or secreted protein involved in the regulation of antitumor immunity, was demonstrated in TILs in pancreatic cancer tissue.

### 3.3 Immunomodulation induced by drugs or X‐ray in pancreatic cancer

Favorable effects might be achieved by removing immunosuppressive mechanisms in pancreatic cancer. Recent studies showed that certain types of chemo‐ and radiotherapy contribute to the activation of antitumor immune response. Cancer cells treated with cyclophosphamide, doxorubicin, oxaliplatin, or mitoxantrone are reported to undergo immunogenic cell death. In addition, 5‐fluorouracil‐ or gemcitabine‐treated cancer cells also become immunogenic.^46^, ^47^, ^48^ Radiotherapy also induces immunogenic cell death. The “abscopal effect”, a phenomenon by which metastatic lesions outside the irradiation field show reductions in size, suggests some form of underlying immunological response.^49^ Homma et al^50^ reported that neoadjuvant chemoradiotherapy (NACRT) consisting of gemcitabine plus S‐1 followed by 30 Gy radiation for pancreatic cancer enhances both CD4^+^ and CD8^+^ TILs. Furthermore, abundant CD8^+^ TILs or scarce Foxp3^+^ TILs after NACRT are associated with favorable long‐term outcomes.^50^, ^51^ MHC class I chain‐related gene A and gene B (MICA/B), a ligand of transmembrane protein, natural killer group 2 member D, is able to activate CD8^+^ T cells and γδT cells, as well as NK cells. Murakami et al showed that damage‐associated molecular patterns (DAMP) such as MICA/B, calreticulin, and heat‐shock protein 70 (Hsp70) were overexpressed after NACRT, and MICA/B was associated with favorable TIL status, suggesting MICA/B as an important regulator of immunomodulation.^51^ Moreover, proteomic analysis of pancreatic cancer treated with NACRT showed that marginal‐zone B‐ and B1‐cell‐specific protein (MZB1) expression was associated with abundant CD8^+^ TIL and longer survival.^52^ These results indicate that immunogenic cell death induced by chemoradiation plays a pivotal role in pancreatic cancer.

## 4 CANCER STROMA‐TARGETING THERAPY FOR PANCREATIC CANCER

Depletion of the desmoplastic stroma has led to better chemotherapy delivery and drug response in preclinical models of pancreatic cancer.^53^, ^54^, ^55^ Antifibrotic therapy therefore appears to represent a promising strategy in the treatment of pancreatic cancer.

Therapeutic strategies to target CAFs in pancreatic cancer include treatments that reduce the abundance of stroma in pancreatic cancer, such as nab‐paclitaxel, pegvorhyaluronidase alfa (PEGPH20), pirfenidone, SOM230 and CD40 agonists,^53^, ^56^, ^57^, ^58^, ^59^ and that reduce CAFs proliferation, including hedgehog pathway inhibitors, multi‐MMP inhibitors, TGF‐β inhibitor, retinoic acid^53^, ^60^, ^61^, ^62^ or vitamin D receptor activation to reprogram CAFs to a quiescent phenotype.^63^

Chemotherapy combining nab‐paclitaxel with gemcitabine has recently become the standard regimen for patients with metastatic pancreatic cancer, significantly prolonging overall survival in the MPACT trial, which was an international, multicenter, open‐label, randomized phase III study.^64^ Exploratory analysis was carried out to gain insight into the role of SPARC expression as a predictor of survival, because nab‐paclitaxel was reported to decrease CAFs and increase microvessel density, attributed to increased drug concentration in tumors treated by nab‐paclitaxel in preclinical models.^65^, ^66^, ^67^ However, stromal and tumor levels of SPARC as measured by immunohistochemistry showed no correlation with overall survival.^68^

Metastatic pancreatic ductal adenocarcinoma (PDAC) is characterized by excessive accumulation of hyaluronan (HA) in the tumor microenvironment, elevating interstitial pressure and impairing perfusion. Preclinical studies have shown that PEGPH20 degrades HA, thereby increasing drug delivery.^53^ A randomized phase II study of PEGPH20 plus nab‐paclitaxel/gemcitabine (PAG) compared with nab‐paclitaxel/gemcitabine (AG) in patients with untreated metastatic pancreatic ductal adenocarcinoma (the HOLO202 trial) showed the largest improvement in progression‐free survival among patients with hihg‐HA tumors who received PAG.^69^

SOM230, a next‐generation somatostatin analogue, resensitized pancreatic cancer cells to chemotherapeutic drugs by inhibiting CAF secretory activity through inhibition of the mammalian target of rapamycin/eukaryotic translation initiation factor 4E binding protein 1 pathway.^58^ SOM230 has already been approved by the Food and Drug Administration for the treatment of Cushing's pituitary tumors, and clinical trials in the setting of pancreatic ductal adenocarcinoma are thus anticipated. In another approach, Beatty et al tested the combination of agonist CD40 antibody with gemcitabine chemotherapy in a small cohort of patients with unresectable PDAC, resulting in tumor regressions in some patients. They demonstrated that CD40‐activated macrophages rapidly infiltrated tumors, which became tumoricidal, and facilitated the depletion of tumor stroma.^59^

The first successful approach to reduce CAF proliferation that led to depletion of tumor stroma and better gemcitabine delivery and prolonging survival in initial preclinical studies, was achieved through inhibition of sonic hedgehog signaling.^54^ The results of that study paved the way for clinical trials. Various hedgehog‐ pathway inhibitors were tested in a phase II trial in the setting of advanced solid tumors, including pancreatic cancer. Unfortunately, this very promising approach in targeting the proliferation of CAFs using hedgehog‐pathway inhibitors failed in phase II trials.^70^ Other preclinical studies indicated that sonic hedgehog signaling inhibition resulted in tumor progression even though desmoplasia was decreased.^71^, ^72^ Moreover, clinical research using pancreatic cancer patient specimens demonstrated that high stromal density was associated with longer survival.^73^ Given these results, part of the components of desmoplastic stroma work as tumor‐restraining rather than as tumor‐promoting.^74^ A similar lesson has been learned from multi‐MMP inhibitors, which did not improve survival among patients with PDAC in clinical trials, despite encouraging preclinical data. Recent data, however, have shown that some MMPs are protective against cancer and others are not, so non‐selective inhibition also cancels the protective effects of some MMPs. Furthermore, initial clinical trials were faulty in that inhibitors were tested in late‐stage cancers, whereas animal data were obtained during cancer initiation. Timing has to be taken into consideration, and entry criteria for clinical trials should be early‐stage of cancer patients in order to match animal data.^60^

In a mouse model, a TGF‐β antagonist suppressed metastasis without any adverse effects.^75^ In another report, TGF‐β inhibition reduced pancreatic cancer stroma in an orthotopic pancreatic‐cancer mouse model, suggesting TGF‐β inhibition as a potential treatment for controlling stroma density.^62^ In a phase Ib clinical trial, the TGF‐β inhibitor galunisertib was given in combination with gemcitabine to patients with advanced or metastatic pancreatic cancer.^76^ The response rate with TGF‐β inhibition therapy was 42.9% with acceptable safety.

## 5 IMMUNOTHERAPY FOR PANCREATIC CANCER

William Coley, known as the father of cancer immunotherapy, noticed that bacterial infection induced notable tumor shrinkage in patients with osteosarcoma. He started bacterial therapy using bacteria or bacterial components, called “Coley's toxin” in 1891, and significant treatment effects were observed in patients with sarcoma.^77^, ^78^, ^79^ The antitumor effect induced by this bacterial therapy is attributable to activation of the immune system followed by inflammation. Although bacterial therapy was replaced with chemotherapy and radiotherapy from the early 20th century, the achievements of bacterial therapy have recently been rediscovered because of marked developments of immunotherapy.

Anti‐CTLA‐4 and anti‐PD‐1 antibody are both immune checkpoint inhibitors that activate tumor‐specific CD8^+^ T‐cell responses. Krummel et al reported CTLA‐4 is a restricting factor for T cells in 1995.^80^ CTLA‐4 antibody therapy was the first immunotherapy drug to significantly prolong overall survival in patients with metastatic melanoma.^81^ In recent years, a new immune checkpoint inhibitor therapy, targeting CTLA‐4 for pancreatic cancer, showed limited efficacy.^21^, ^82^, ^83^, ^84^ However, in a tumor microenvironment in which immune reaction has been stimulated, checkpoint inhibition may be more effective. Tumeh et al^85^ reported that tumor response to anti‐programmed cell death protein 1 (anti‐PD‐1) therapy depended on pre‐existing CD8^+^ TILs in melanoma patients. Considering that CD8^+^ TILs in pancreatic cancer stroma were induced by NACRT, PD‐1 blockade may be effective in combination with NACRT. In addition, programmed death‐ligand 1 (PD‐L1)‐positive responses in more than half of the cancer cells within tumors indicated good response to PD‐1 inhibitor therapy in patients with non‐small cell lung cancer.^86^ PD‐L1 overexpression in pancreatic cancer cells is thus possibly predictive of the response to anti‐PD‐1 therapy.

Tumor‐infiltrating lymphocyte adoptive cell therapy has been developing since the 1980s. TILs extracted from resected specimens were stimulated and cultured in vitro, then transfused into patients. Rosenberg and Restifo^87^ reported that the objective response rate for TIL adoptive cell therapy in melanoma patients ranged from 34% to 56%. Although the efficacy of TIL adoptive cell therapy for pancreatic cancer has not yet been reported,^88^ this immunotherapy appears to have functional potential as TILs are likely to be a prognostic factor. Sakellariou‐Thompson et al^89^ recently noted that CD8^+^ TILs derived from pancreatic cancer tissue could be grown with the aid of a 4‐1BB agonist, suggesting the clinical feasibility of TIL adoptive cell therapy.

Chimeric antigen receptor T‐cell (CAR‐T) therapy has shown high remission rate for patients with acute lymphoblastic leukemia.^90^ Cultured T cells transferred with the CAR gene using a retroviral or lentiviral vector are reinjected into the host. CAR‐T therapy directly stimulates cell‐mediated immunity, and can thus induce stronger antitumor immune reaction than antibody therapy.^91^ Several studies of CAR‐T therapy for pancreatic cancer are under way.^88^

Targeting immunosuppressive cells may be promising. Tumor‐infiltrating Tregs in melanoma patients highly express chemokine receptor type 4 (CCR4), a potential target for Treg depletion. CCR4 antibody has been shown to remove effector‐type Treg both in vivo and in vitro.^92^ Mogamulizumab, a humanized anti‐CCR4 antibody therapy for solid tumors, is under clinical study.^93^

Bacterial therapy may become a potential immunotherapy for pancreatic cancer. *Salmonella typhimurium* A1‐R has been shown to be effective in patient‐derived xenograft mouse models of pancreatic cancer.^94^ In addition, *S*.* typhimurium* A1‐R enhanced CD8^+^ TILs in a syngeneic pancreatic cancer mouse model, suggesting activation of host antitumor immunity.^95^

## 6 CONCLUSIONS

The tumor microenvironment in pancreatic cancer contributes to tumor growth, invasion, and metastasis in a multifaceted way, including immune evasion. New immunotherapies or cancer stroma‐targeting therapies have potential to induce a cure for pancreatic cancer.

## DISCLOSURE

Conflicts of Interest: Authors declare no conflicts of interest for this article.

## References

[1] Siegel RL , Miller KD , Jemal A . Cancer Statistics, 2017. CA Cancer J Clin. 2017;67:7–30. 28055103 10.3322/caac.21387

[2] Rahib L , Smith BD , Aizenberg R , Rosenzweig AB , Fleshman JM , Matrisian LM , et al. Projecting cancer incidence and deaths to 2030: the unexpected burden of thyroid, liver, and pancreas cancers in the United States. Cancer Res. 2014;74:2913–21. 24840647 10.1158/0008-5472.CAN-14-0155

[3] Karagiannis GS , Poutahidis T , Erdman SE , Kirsch R , Riddell RH , Diamandis EP , et al. Cancer‐associated fibroblasts drive the progression of metastasis through both paracrine and mechanical pressure on cancer tissue. Mol Cancer Res. 2012;10:1403–18. 23024188 10.1158/1541-7786.MCR-12-0307 PMC4399759

[4] Wu J , Liang C , Chen M , Su W . Association between tumor‐stroma ratio and prognosis in solid tumor patients: a systematic review and meta‐analysis. Oncotarget. 2016;7:68954–65. 27661111 10.18632/oncotarget.12135 PMC5356603

[5] Hidalgo M . Pancreatic cancer. N Engl J Med. 2010;362:1605–17. 20427809 10.1056/NEJMra0901557

[6] Whatcott C , Han H , Posner RG , Von Hoff DD . Tumor‐stromal interactions in pancreatic cancer. Crit Rev Oncog. 2013;18:135–51. 23237556 10.1615/critrevoncog.v18.i1-2.80 PMC3632415

[7] Bi S , Wang Y , Guan J , Sheng X , Meng J . Three new Jurassic euharamiyidan species reinforce early divergence of mammals. Nature. 2014;514:579–84. 25209669 10.1038/nature13718

[8] Giancotti FG , Ruoslahti E . Integrin signaling. Science. 1999;285:1028–32. 10446041 10.1126/science.285.5430.1028

[9] Whatcott CJ , Han H , Posner RG , Hostetter G , Von Hoff DD . Targeting the tumor microenvironment in cancer: why hyaluronidase deserves a second look. Cancer Discov. 2011;1:291–6. 22053288 10.1158/2159-8290.CD-11-0136 PMC3204883

[10] Mace TA , Ameen Z , Collins A , et al. Pancreatic cancer‐associated stellate cells promote differentiation of myeloid‐derived suppressor cells in a STAT3‐dependent manner. Cancer Res. 2013;73:3007–18. 23514705 10.1158/0008-5472.CAN-12-4601 PMC3785672

[11] von Ahrens D , Bhagat TD , Nagrath D , Maitra A , Verma A . The role of stromal cancer‐associated fibroblasts in pancreatic cancer. J Hematol Oncol. 2017;10:76. 28351381 10.1186/s13045-017-0448-5 PMC5371211

[12] Shan T , Chen S , Chen X , et al. Cancer‐associated fibroblasts enhance pancreatic cancer cell invasion by remodeling the metabolic conversion mechanism. Oncol Rep. 2017;37:1971–9. 28260082 10.3892/or.2017.5479 PMC5367364

[13] De Wever O , Westbroek W , Verloes A , et al. Critical role of N‐cadherin in myofibroblast invasion and migration in vitro stimulated by colon‐cancer‐cell‐derived TGF‐beta or wounding. J Cell Sci. 2004;117:4691–703. 15331629 10.1242/jcs.01322

[14] Naber HP , ten Dijke P , Pardali E . Role of TGF‐beta in the tumor stroma. Curr Cancer Drug Targets. 2008;8:466–72. 18781893 10.2174/156800908785699342

[15] Bierie B , Moses HL . Tumour microenvironment: TGFbeta: the molecular Jekyll and Hyde of cancer. Nat Rev Cancer. 2006;6:506–20. 16794634 10.1038/nrc1926

[16] Togo S , Polanska UM , Horimoto Y , Orimo A . Carcinoma‐associated fibroblasts are a promising therapeutic target. Cancers (Basel). 2013;5:149–69. 24216702 10.3390/cancers5010149 PMC3730310

[17] De Wever O , Pauwels P , De Craene B , et al. Molecular and pathological signatures of epithelial‐mesenchymal transitions at the cancer invasion front. Histochem Cell Biol. 2008;130:481–94. 18648847 10.1007/s00418-008-0464-1 PMC2522326

[18] Chang JH , Jiang Y , Pillarisetty VG . Role of immune cells in pancreatic cancer from bench to clinical application: an updated review. Medicine (Baltimore). 2016;95:e5541. 27930550 10.1097/MD.0000000000005541 PMC5266022

[19] Ryschich E , Notzel T , Hinz U , et al. Control of T‐cell‐mediated immune response by HLA class I in human pancreatic carcinoma. Clin Cancer Res. 2005;11:498–504. 15701833

[20] Schumacher TN , Schreiber RD . Neoantigens in cancer immunotherapy. Science. 2015;348:69–74. 25838375 10.1126/science.aaa4971

[21] Bailey P , Chang DK , Forget MA , et al. Exploiting the neoantigen landscape for immunotherapy of pancreatic ductal adenocarcinoma. Sci Rep. 2016;6:35848. 27762323 10.1038/srep35848 PMC5071896

[22] Moo‐Young TA , Larson JW , Belt BA , et al. Tumor‐derived TGF‐beta mediates conversion of CD4 + Foxp3 + regulatory T cells in a murine model of pancreas cancer. J Immunother. 2009;32:12–21. 19307989 10.1097/CJI.0b013e318189f13c PMC3862184

[23] Bellone G , Turletti A , Artusio E , et al. Tumor‐associated transforming growth factor‐beta and interleukin‐10 contribute to a systemic Th2 immune phenotype in pancreatic carcinoma patients. Am J Pathol. 1999;155:537–47. 10433946 10.1016/s0002-9440(10)65149-8 PMC1866873

[24] Uyttenhove C , Pilotte L , Theate I , et al. Evidence for a tumoral immune resistance mechanism based on tryptophan degradation by indoleamine 2,3‐dioxygenase. Nat Med. 2003;9:1269–74. 14502282 10.1038/nm934

[25] Clark CE , Hingorani SR , Mick R , Combs C , Tuveson DA , Vonderheide RH . Dynamics of the immune reaction to pancreatic cancer from inception to invasion. Cancer Res. 2007;67:9518–27. 17909062 10.1158/0008-5472.CAN-07-0175

[26] Sakaguchi S , Sakaguchi N , Asano M , Itoh M , Toda M . Immunologic self‐tolerance maintained by activated T cells expressing IL‐2 receptor alpha‐chains (CD25). Breakdown of a single mechanism of self‐tolerance causes various autoimmune diseases. J Immunol 1995;155:1151–64. 7636184

[27] Hori S , Nomura T , Sakaguchi S . Pillars article: control of regulatory T cell development by the transcription factor Foxp3. Science 2003. 299: 1057‐1061. J Immunol 2017; 198: 981–5. 28115586

[28] Nishikawa H , Sakaguchi S . Regulatory T cells in cancer immunotherapy. Curr Opin Immunol. 2014;27:1–7. 24413387 10.1016/j.coi.2013.12.005

[29] Homma Y , Taniguchi K , Nakazawa M , et al. Changes in the immune cell population and cell proliferation in peripheral blood after gemcitabine‐based chemotherapy for pancreatic cancer. Clin Transl Oncol. 2014;16:330–5. 23860726 10.1007/s12094-013-1079-0

[30] Vignali DA , Collison LW , Workman CJ . How regulatory T cells work. Nat Rev Immunol. 2008;8:523–32. 18566595 10.1038/nri2343 PMC2665249

[31] Gabitass RF , Annels NE , Stocken DD , Pandha HA , Middleton GW . Elevated myeloid‐derived suppressor cells in pancreatic, esophageal and gastric cancer are an independent prognostic factor and are associated with significant elevation of the Th2 cytokine interleukin‐13. Cancer Immunol Immunother. 2011;60:1419–30. 21644036 10.1007/s00262-011-1028-0 PMC3176406

[32] Murdoch C , Giannoudis A , Lewis CE . Mechanisms regulating the recruitment of macrophages into hypoxic areas of tumors and other ischemic tissues. Blood. 2004;104:2224–34. 15231578 10.1182/blood-2004-03-1109

[33] Noy R , Pollard JW . Tumor‐associated macrophages: from mechanisms to therapy. Immunity. 2014;41:49–61. 25035953 10.1016/j.immuni.2014.06.010 PMC4137410

[34] Allavena P , Piemonti L , Longoni D , et al. IL‐10 prevents the differentiation of monocytes to dendritic cells but promotes their maturation to macrophages. Eur J Immunol. 1998;28:359–69. 9485215 10.1002/(SICI)1521-4141(199801)28:01<359::AID-IMMU359>3.0.CO;2-4

[35] Peranzoni E , Lemoine J , Vimeux L , et al. Macrophages impede CD8 T cells from reaching tumor cells and limit the efficacy of anti‐PD‐1 treatment. Proc Natl Acad Sci USA. 2018;115:E4041–50. 29632196 10.1073/pnas.1720948115 PMC5924916

[36] Matsushita H , Vesely MD , Koboldt DC , et al. Cancer exome analysis reveals a T‐cell‐dependent mechanism of cancer immunoediting. Nature. 2012;482:400–4. 22318521 10.1038/nature10755 PMC3874809

[37] Zito Marino F , Ascierto PA , Rossi G , et al. Are tumor‐infiltrating lymphocytes protagonists or background actors in patient selection for cancer immunotherapy? Expert Opin Biol Ther. 2017;17:735–46. 28318336 10.1080/14712598.2017.1309387

[38] Solinas C , Pusole G , Demurtas L , et al. Tumor infiltrating lymphocytes in gastrointestinal tumors: Controversies and future clinical implications. Crit Rev Oncol Hematol. 2017;110:106–16. 28109400 10.1016/j.critrevonc.2016.11.016

[39] Fukunaga A , Miyamoto M , Cho Y , et al. CD8+ tumor‐infiltrating lymphocytes together with CD4+ tumor‐infiltrating lymphocytes and dendritic cells improve the prognosis of patients with pancreatic adenocarcinoma. Pancreas. 2004;28:e26–31. 14707745 10.1097/00006676-200401000-00023

[40] Hwang HK , Kim HI , Kim SH , et al. Prognostic impact of the tumor‐infiltrating regulatory T‐cell (Foxp3(+))/activated cytotoxic T lymphocyte (granzyme B(+)) ratio on resected left‐sided pancreatic cancer. Oncol Lett. 2016;12:4477–84. 28105157 10.3892/ol.2016.5252 PMC5228542

[41] Zhan HX , Zhou B , Cheng YG , et al. Crosstalk between stromal cells and cancer cells in pancreatic cancer: new insights into stromal biology. Cancer Lett. 2017;392:83–93. 28189533 10.1016/j.canlet.2017.01.041

[42] Hu G , Wang S , Cheng P . Tumor‐infiltrating tryptase(+) mast cells predict unfavorable clinical outcome in solid tumors. Int J Cancer. 2018;142:813–21. 29023696 10.1002/ijc.31099

[43] Strouch MJ , Cheon EC , Salabat MR , et al. Crosstalk between mast cells and pancreatic cancer cells contributes to pancreatic tumor progression. Clin Cancer Res. 2010;16:2257–65. 20371681 10.1158/1078-0432.CCR-09-1230 PMC3122919

[44] Suzuki S , Ichikawa Y , Nakagawa K , et al. High infiltration of mast cells positive to tryptase predicts worse outcome following resection of colorectal liver metastases. BMC Cancer. 2015;15:840. 26530140 10.1186/s12885-015-1863-z PMC4632336

[45] Kato S , Kubota K , Shimamura T , et al. Semaphorin 4D, a lymphocyte semaphorin, enhances tumor cell motility through binding its receptor, plexinB1, in pancreatic cancer. Cancer Sci. 2011;102:2029–37. 21812859 10.1111/j.1349-7006.2011.02053.x

[46] Gelbard A , Garnett CT , Abrams SI , et al. Combination chemotherapy and radiation of human squamous cell carcinoma of the head and neck augments CTL‐mediated lysis. Clin Cancer Res. 2006;12:1897–905. 16551875 10.1158/1078-0432.CCR-05-1761 PMC1865094

[47] Tomihara K , Fuse H , Heshiki W , et al. Gemcitabine chemotherapy induces phenotypic alterations of tumor cells that facilitate antitumor T cell responses in a mouse model of oral cancer. Oral Oncol. 2014;50:457–67. 24582211 10.1016/j.oraloncology.2014.01.013

[48] Vacchelli E , Galluzzi L , Fridman WH , et al. Trial watch: chemotherapy with immunogenic cell death inducers. Oncoimmunology. 2012;1:179–88. 22720239 10.4161/onci.1.2.19026 PMC3376992

[49] Golden EB , Apetoh L . Radiotherapy and immunogenic cell death. Semin Radiat Oncol. 2015;25:11–7. 25481261 10.1016/j.semradonc.2014.07.005

[50] Homma Y , Taniguchi K , Murakami T , et al. Immunological impact of neoadjuvant chemoradiotherapy in patients with borderline resectable pancreatic ductal adenocarcinoma. Ann Surg Oncol. 2014;21:670–6. 24310792 10.1245/s10434-013-3390-y

[51] Murakami T , Homma Y , Matsuyama R , et al. Neoadjuvant chemoradiotherapy of pancreatic cancer induces a favorable immunogenic tumor microenvironment associated with increased major histocompatibility complex class I‐related chain A/B expression. J Surg Oncol. 2017;116:416–26. 28608409 10.1002/jso.24681

[52] Miyake K , Mori R , Homma Y , et al. MZB1 in borderline resectable pancreatic cancer resected after neoadjuvant chemoradiotherapy. J Surg Res. 2017;220:391–401. 29180208 10.1016/j.jss.2017.07.003

[53] Jacobetz MA , Chan DS , Neesse A , et al. Hyaluronan impairs vascular function and drug delivery in a mouse model of pancreatic cancer. Gut. 2013;62:112–20. 22466618 10.1136/gutjnl-2012-302529 PMC3551211

[54] Olive KP , Jacobetz MA , Davidson CJ , et al. Inhibition of Hedgehog signaling enhances delivery of chemotherapy in a mouse model of pancreatic cancer. Science. 2009;324:1457–61. 19460966 10.1126/science.1171362 PMC2998180

[55] Provenzano PP , Cuevas C , Chang AE , Goel VK , Von Hoff DD , Hingorani SR . Enzymatic targeting of the stroma ablates physical barriers to treatment of pancreatic ductal adenocarcinoma. Cancer Cell. 2012;21:418–29. 22439937 10.1016/j.ccr.2012.01.007 PMC3371414

[56] Kozono S , Ohuchida K , Eguchi D , et al. Pirfenidone inhibits pancreatic cancer desmoplasia by regulating stellate cells. Cancer Res. 2013;73:2345–56. 23348422 10.1158/0008-5472.CAN-12-3180

[57] Giordano G , Pancione M , Olivieri N , et al. Nano albumin bound‐paclitaxel in pancreatic cancer: current evidences and future directions. World J Gastroenterol. 2017;23:5875–86. 28932079 10.3748/wjg.v23.i32.5875 PMC5583572

[58] Duluc C , Moatassim‐Billah S , Chalabi‐Dchar M , et al. Pharmacological targeting of the protein synthesis mTOR/4E‐BP1 pathway in cancer‐associated fibroblasts abrogates pancreatic tumour chemoresistance. EMBO Mol Med. 2015;7:735–53. 25834145 10.15252/emmm.201404346 PMC4459815

[59] Beatty GL , Chiorean EG , Fishman MP , et al. CD40 agonists alter tumor stroma and show efficacy against pancreatic carcinoma in mice and humans. Science. 2011;331:1612–6. 21436454 10.1126/science.1198443 PMC3406187

[60] Amar S , Fields GB . Potential clinical implications of recent matrix metalloproteinase inhibitor design strategies. Expert Rev Proteomics. 2015;12:445–7. 26174966 10.1586/14789450.2015.1069190 PMC4829401

[61] Froeling FE , Feig C , Chelala C , et al. Retinoic acid‐induced pancreatic stellate cell quiescence reduces paracrine Wnt‐beta‐catenin signaling to slow tumor progression. Gastroenterology. 2011;141(1486–1497):1497.e1–14. 10.1053/j.gastro.2011.06.047 21704588

[62] Murakami T , Hiroshima Y , Miyake K , et al. Color‐coded intravital imaging demonstrates a transforming growth factor‐beta (TGF‐beta) antagonist selectively targets stromal cells in a human pancreatic‐cancer orthotopic mouse model. Cell Cycle. 2017;16:1008–14. 28441080 10.1080/15384101.2017.1315489 PMC5462077

[63] Sherman MH , Yu RT , Engle DD , et al. Vitamin D receptor‐mediated stromal reprogramming suppresses pancreatitis and enhances pancreatic cancer therapy. Cell. 2014;159:80–93. 25259922 10.1016/j.cell.2014.08.007 PMC4177038

[64] Von Hoff DD , Ervin T , Arena FP , et al. Increased survival in pancreatic cancer with nab‐paclitaxel plus gemcitabine. N Engl J Med. 2013;369:1691–703. 24131140 10.1056/NEJMoa1304369 PMC4631139

[65] Rajeshkumar NV , Yabuuchi S , Pai SG , et al. Superior therapeutic efficacy of nab‐paclitaxel over cremophor‐based paclitaxel in locally advanced and metastatic models of human pancreatic cancer. Br J Cancer. 2016;115:442–53. 27441498 10.1038/bjc.2016.215 PMC4985357

[66] Alvarez R , Musteanu M , Garcia‐Garcia E , et al. Stromal disrupting effects of nab‐paclitaxel in pancreatic cancer. Br J Cancer. 2013;109:926–33. 23907428 10.1038/bjc.2013.415 PMC3749580

[67] Suenaga M , Yamada S , Fujii T , et al. S‐1 plus nab‐paclitaxel is a promising regimen for pancreatic cancer in a preclinical model. J Surg Oncol. 2016;113:413–9. 27100026 10.1002/jso.24147

[68] Hidalgo M , Plaza C , Musteanu M , et al. SPARC expression did not predict efficacy of nab‐paclitaxel plus gemcitabine or gemcitabine alone for metastatic pancreatic cancer in an exploratory analysis of the phase III MPACT trial. Clin Cancer Res. 2015;21:4811–8. 26169969 10.1158/1078-0432.CCR-14-3222

[69] Hingorani SR , Zheng L , Bullock AJ , et al. HALO 202: randomized phase II study of PEGPH20 Plus nab‐paclitaxel/gemcitabine versus nab‐paclitaxel/gemcitabine in patients with untreated, metastatic pancreatic ductal adenocarcinoma. J Clin Oncol. 2018;36:359–66. 29232172 10.1200/JCO.2017.74.9564

[70] Amakye D , Jagani Z , Dorsch M . Unraveling the therapeutic potential of the Hedgehog pathway in cancer. Nat Med. 2013;19:1410–22. 24202394 10.1038/nm.3389

[71] Rhim AD , Oberstein PE , Thomas DH , et al. Stromal elements act to restrain, rather than support, pancreatic ductal adenocarcinoma. Cancer Cell. 2014;25:735–47. 24856585 10.1016/j.ccr.2014.04.021 PMC4096698

[72] Lee JJ , Perera RM , Wang H , et al. Stromal response to Hedgehog signaling restrains pancreatic cancer progression. Proc Natl Acad Sci USA. 2014;111:E3091–100. 25024225 10.1073/pnas.1411679111 PMC4121834

[73] Bever KM , Sugar EA , Bigelow E , et al. The prognostic value of stroma in pancreatic cancer in patients receiving adjuvant therapy. HPB (Oxford). 2015;17:292–8. 25250696 10.1111/hpb.12334 PMC4368391

[74] Gu J , Saiyin H , Fu D , Li J . Stroma ‐ a double‐edged sword in pancreatic cancer: a lesson from targeting stroma in pancreatic cancer with Hedgehog signaling inhibitors. Pancreas. 2018;47:382–9. 29521941 10.1097/MPA.0000000000001023

[75] Yang Y‐A , Dukhanina O , Tang B , et al. Lifetime exposure to a soluble TGF‐β antagonist protects mice against metastasis without adverse side effects. J Clin Invest 2002; 109: 1607–15. 12070308 10.1172/JCI15333 PMC151015

[76] Ikeda M , Takahashi H , Kondo S , et al. Phase 1b study of galunisertib in combination with gemcitabine in Japanese patients with metastatic or locally advanced pancreatic cancer. Cancer Chemother Pharmacol. 2017;79:1169–77. 28451833 10.1007/s00280-017-3313-x

[77] Nauts HC , Swift WE , Coley BL . The treatment of malignant tumors by bacterial toxins as developed by the late William B. Coley, M.D., reviewed in the light of modern research. Cancer Res 1946; 6:205–16. 21018724

[78] Wolchok JD , Chan TA . Cancer: antitumour immunity gets a boost. Nature. 2014;515:496–8. 25428495 10.1038/515496a PMC6592276

[79] McCarthy EF . The toxins of William B Coley and the treatment of bone and soft‐tissue sarcomas. Iowa Orthop J 2006;26:154–8. 16789469 PMC1888599

[80] Krummel MF , Allison JP . CD28 and CTLA‐4 have opposing effects on the response of T cells to stimulation. J Exp Med. 1995;182:459–65. 7543139 10.1084/jem.182.2.459 PMC2192127

[81] Hodi FS , O'Day SJ , McDermott DF , et al. Improved survival with ipilimumab in patients with metastatic melanoma. N Engl J Med. 2010;363:711–23. 20525992 10.1056/NEJMoa1003466 PMC3549297

[82] Johansson H , Andersson R , Bauden M , Hammes S , Holdenrieder S , Ansari D . Immune checkpoint therapy for pancreatic cancer. World J Gastroenterol. 2016;22:9457–76. 27920468 10.3748/wjg.v22.i43.9457 PMC5116591

[83] Aglietta M , Barone C , Sawyer MB , et al. A phase I dose escalation trial of tremelimumab (CP‐675,206) in combination with gemcitabine in chemotherapy‐naive patients with metastatic pancreatic cancer. Ann Oncol. 2014;25:1750–5. 24907635 10.1093/annonc/mdu205

[84] Royal RE , Levy C , Turner K , et al. Phase 2 trial of single agent Ipilimumab (anti‐CTLA‐4) for locally advanced or metastatic pancreatic adenocarcinoma. J Immunother. 2010;33:828–33. 20842054 10.1097/CJI.0b013e3181eec14c PMC7322622

[85] Tumeh PC , Harview CL , Yearley JH , et al. PD‐1 blockade induces responses by inhibiting adaptive immune resistance. Nature. 2014;515:568–71. 25428505 10.1038/nature13954 PMC4246418

[86] Herbst RS , Baas P , Kim D‐W , et al. Pembrolizumab versus docetaxel for previously treated, PD‐L1‐positive, advanced non‐small‐cell lung cancer (KEYNOTE‐010): a randomised controlled trial. Lancet. 2016;387:1540–50. 26712084 10.1016/S0140-6736(15)01281-7

[87] Rosenberg SA , Restifo NP . Adoptive cell transfer as personalized immunotherapy for human cancer. Science. 2015;348:62–8. 25838374 10.1126/science.aaa4967 PMC6295668

[88] Liu F , Saif MW . T cell optimization for the treatment of pancreatic cancer. Expert Opin Biol Ther. 2017;17:1493–501. 28835191 10.1080/14712598.2017.1369948

[89] Sakellariou‐Thompson D , Forget MA , Creasy C , et al. 4‐1BB agonist focuses CD8(+) tumor‐infiltrating T‐cell growth into a distinct repertoire capable of tumor recognition in pancreatic cancer. Clin Cancer Res. 2017;23:7263–75. 28947567 10.1158/1078-0432.CCR-17-0831 PMC6097625

[90] Frey NV , Porter DL . CAR T‐cells merge into the fast lane of cancer care. Am J Hematol. 2016;91:146–50. 26574400 10.1002/ajh.24238

[91] Almasbak H , Aarvak T , Vemuri MC . CAR T cell therapy: a game changer in cancer treatment. J Immunol Res. 2016;2016:5474602. 27298832 10.1155/2016/5474602 PMC4889848

[92] Sugiyama D , Nishikawa H , Maeda Y , et al. Anti‐CCR4 mAb selectively depletes effector‐type FoxP3+CD4+ regulatory T cells, evoking antitumor immune responses in humans. Proc Natl Acad Sci USA. 2013;110:17945–50. 24127572 10.1073/pnas.1316796110 PMC3816454

[93] Kurose K , Ohue Y , Wada H , et al. Phase Ia study of FoxP3+ CD4 Treg depletion by infusion of a humanized anti‐CCR4 antibody, KW‐0761, in cancer patients. Clin Cancer Res. 2015;21:4327–36. 26429981 10.1158/1078-0432.CCR-15-0357

[94] Hiroshima Y , Zhang Y , Murakami T , et al. Efficacy of tumor‐targeting *Salmonella typhimurium* A1‐R in combination with anti‐angiogenesis therapy on a pancreatic cancer patient‐derived orthotopic xenograft (PDOX) and cell line mouse models. Oncotarget. 2014;5:12346–57. 25402324 10.18632/oncotarget.2641 PMC4322966

[95] Murakami T , Hiroshima Y , Zhang Y , et al. Tumor‐targeting *Salmonella typhimurium* A1‐R promotes tumoricidal CD8(+) T cell tumor infiltration and arrests growth and metastasis in a syngeneic pancreatic‐cancer orthotopic mouse model. J Cell Biochem. 2018;119:634–9. 28628234 10.1002/jcb.26224

